# Book Notices

**Published:** 1885-10

**Authors:** 


### BOOK NOTICES.

DOMESTIC GUIDE TO THE HOMŒOPATHIC TREATMENT, and also the Hygienic Measures Required in the Management, of Epidemic Cholera. By J. A. Biegler, M. D., Rochester, N. Y.

Though this little work is intended solely for domestic use, the professional man will be amply rewarded by its perusal. A brief history of cholera, in connection with homœopathic therapeutics, is given, and complete evidence adduced of the grand success Homœopathy has won against this dread enemy.

HOW TO SEE WITH THE MICROSCOPE, etc., etc. By J. Edwards Smith, M. D. Second edition; Chicago: Duncan Brothers, 1885.

In our notice of the first edition of Dr. Smith's work a description of its purposes and character was given. It is not necessary at this time to repeat that description. We may simply add that the second edition is enlarged, revised, and improved. The purpose of the book is to teach one how to use a microscope. This is very well done. The book is indispensable to those learning to use that valuable, now almost indispensable, instrument, the microscope.

LECTURES ON CLINICAL OTOLOGY. By Henry C. Houghton, M. D. Pp. 260. Boston: Otis Clapp & Son. 1885.

This volume consists of twelve lectures, delivered before the senior class in the New York Homœopathic Medical College, to which are added cases from practice and summaries of remedies; also, an appendix with a useful repertory of symptoms appertaining to the ear. The first lecture is introductory and good; those succeeding treat of the more common ailments of the external, middle, and internal ear; such as inflammation, catarrhs, ulcers, morbid growths, etc., the last lecture being on deaf-mutism and helps to hearing.

Dr. Houghton has acquired a valuable reputation for his skill in treating diseases of the ear. This volume gives evidence of this skill. The work is well written, well illustrated, and the therapeutic measures recommended are in the main such as homœopathists find useful. In some cases topical treatment is recommended and declared to be necessary. The older (and more skillful?) homœopathists declare they have never used, and never needed, *any* kind of topical treatment! Why then do the younger generation need them? Shall we acknowledge ourselves to be less skillful than they of olden time?

A CHART OF TUMORS. By G. F. Shears, M. D. Chicago: 1885.

This chart embraces the classification, characteristics, diagnostic features, prognosis, and treatment of solid and cystic tumors. It is printed on heavy card-board, forming a chart of about two feet square. The whole surface being before the eye at once, it is easily consulted, and comparisons readily made.

SPECIAL PATHOLOGY AND DIAGNOSTICS, WITH THERAPEUTIC HINTS. By Charles G. Raue, M. D. Third Edition. Pp. 1049, 8vo. Half morocco. Philadelphia: F. E. Boericke. 1885.

The third edition of this valuable work has just been received; it is thoroughly revised and considerably enlarged. In addition to revisions and changes, this edition has been considerably enhanced in value by the addition, to the principal chapters, of a "digest" of the remedies recommended—a brief Repertory. This is a valuable feature, and will be appreciated by the practitioner.

The concise, pregnant style which distinguishes all Raue's writings is retained in this new edition, and the volume is a decided improvement on previous editions, which were all good in their day. Dr. Raue's reputation is a sufficient guarantee for the accuracy and usefulness of the work. We can only add: study it.

### A System of Medicine. Based upon the Law of Homœopathy. Edited by H. R. Arndt, M. D. Vol. II. Philadelphia: F. E. Boericke. 1885. Pp. 923, 8vo.

As previously noticed, the second volume of Dr. Arndt's work has just been received. It is gotten up in the best style possible.

A glance at the list of contributors shows that, as in the previous volume, all grades of practitioners have written for this volume. And hence the methods of practice advocated are various and some very dubious. In alphabetical order we find the following writers: H. R. Arndt, M. D., of Grand Rapids; F. E. Doughty, M. D., of New York City; H. B. Fellows, M. D., of Chicago; E. C. Franklin, M. D., of St. Louis; Charles Gatschell, M. D., of Chicago; J. S. Gilchrist, M. D., of Iowa City; W. C. Goodno, M. D., of Philadelphia; G. A. Hull, M. D., of Chicago; Charles Porter Hart, M. D., of Wyoming, O.; J. Martine Kershaw, M. D., of St. Louis; F. Park Lewis, M. D., of Buffalo; S. Lilienthal, M. D., of New York; R. Ludlam, M. D., of Chicago; J. H. McClelland, M. D., of Pittsburgh; J. T. O'Connor, M. D., of Amenia, N. Y.; Julia Holmes Smith, M. D., of Chicago; W. B. Trites, M. D., of Manayunk, Pa; George William Winterburn, M. D., of New York; Samuel Worcester, M. D., of Salem, Mass.

Among these authors are some of the ablest men in the homœopathic ranks, and the list on the whole comprises greater talent than that of the first volume. Some of these gentlemen have given very fine articles on the subjects written of: as was to be expected from their reputation.

But, on the whole, the volume is weakest *where it should be strongest*, *i. e.*, in its therapeutics. In some cases it is not only weak, but absolutely out-and-out eclectic! Thus, p. 508, an ointment of "one drachm of Belladonna to two ounces of lard" is recommended for use in "chronic vaginitis"; it is also stated that "great relief may be obtained from an injection into the vagina of fifteen grains of chloral hydrate to an ounce of water three or four times a day." If such practice be "based on the law of Homœopathy," an explanation of it would be interesting. Contrast such practice with this good counsel, as found on p. 441: "Above all, with the greatest care study out the key-notes of the case;" that advice comprises "the law of Homœopathy" in its strength, and, if followed, will give the greatest success. The adoption of worn-out allopathic expedients does not bring one much success.

The subjects treated of in this volume are the diseases of the spleen, kidneys, ureters, bladder; genital organs, male and female; of the nervous system, general and special; of the locomotory organs, including gout, rheumatism, etc., etc.—subjects full of interest and importance to all practitioners.

We feel justified in believing that these volumes will be of more use to the homœopathic practitioner than the works of Pepper and Ziemssen. The practice recommended is, in many cases, entirely eclectic, and in other cases the treatment as given is meagre and unsatisfactory. Yet what there is that *is good* is worth the entire cost of the volume. The physician should know how to separate the wheat from the tares.

